# Root Canal Morphology of Permanent Mandibular Premolars in Iranian Population: A Systematic Review

**DOI:** 10.7508/iej.2016.03.001

**Published:** 2016-05-01

**Authors:** Sepanta Hosseinpour, Mohammad Javad Kharazifard, Akbar Khayat, Mandana Naseri

**Affiliations:** a *Students' Research Office, Dental School, Shahid Beheshti University of Medical Sciences, Tehran, Iran; *; b * Dental Research Center, Tehran University of Medical Sciences, Tehran, Iran; *; c *Division of Endodontics, Department of Oral Biological and Medical Sciences, Faculty of Dentistry, University of British Columbia, Canada; *; d * Department of Endodontics, Dental School, Shahid Beheshti University of Medical Sciences, Tehran, Iran. *

**Keywords:** Anatomy, Iranian, Mandibular Premolar, Review, Root Canal

## Abstract

**Introduction:**

It is essential for clinicians to have knowledge about root canal configuration, although its morphology varies largely in different ethnicities and even in different individuals within the same ethnic group. The current study reviewed the root canal configuration of root canals in mandibular first and second premolars among Iranian population based on independent epidemiological studies.

**Methods and Materials:**

A comprehensive search was conducted on retrieved articles related to root canal configuration and prevalence of each types of root canal in mandibular premolars based on Vertucci’s classification. An electronic search was conducted in Medline, Scopus and Google Scholar from January 1984 to September 2015.

**Results:**

In eleven studies conducted in eight provinces, 1644 mandibular first premolars and 1268 second premolars were investigated. Within mandibular first premolars, 70.9% were Vertucci's* type I*, followed by 10.4% *type III*, 7.18% *type IV*, 5.23% *type II* and 5.16% *type V*. In addition, among mandibular second premolars, 82.86% were *type I*, 6.25 *type III*, 5.32% *type II*, 4.27% *type IV*, and 0.69% *type V*.

**Conclusion:**

These results highlight the necessity of searching for additional possible root canals by clinicians. Moreover, these results indicated the ethnical characteristics of Iranian population regarding the morphology of mandibular premolars compared to other populations.

## Introduction

Prosperous nonsurgical endodontic therapy is closely associated with locating all root canals, proper mechanical and chemical cleaning and shaping of all root canals, and finally perfect obturation using appropriate sealants and materials [1-5]. Therefore, it is essential for clinicians to have knowledge about root canal configurations although its morphology varies largely in different ethnicities and even in different individuals with the same ethnic [6-8]. The root canal configuration is usually complicated and various [1, 9, 10]. Based on the literature in addition to ethnicity, age [11-13] and gender [14-16] also can influence these diversities. 

**Figure 1:**
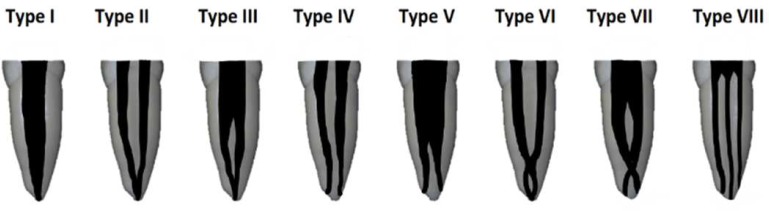
Vertucci’s classification of root canal configuration [17]

Previous studies classified root canal morphology in various ways [12, 17, 18]. First of all, in 1902 GV Black [19] mentioned human tooth morphology. Weine *et al.* [18] in 1969 described a four-type classification method based on the pattern of division of the main root canal. Details of human root canals have been studied by Vertucci [17] in 1984. Vertucci introduced a standardized and categorized method for differentiating root canal variations into the eight descriptive types (Figure 1) [17]. This classification has been widely used in many studies [3, 6, 7, 20-24]. Fourteen new canal morphology types were added to previous classifications [16]. However, many case reports indicate several variations that emphasis on complete evaluation of each case [25-27]. These variations make it difficult to locate, clean and fill all root canals which can lead to post-treatment disease and influence the outcome of root canal therapy [28-30].

Mandibular first and second premolars are typically described as single rooted teeth with ovoid roots in cross sections and developmental concavities on mesial and distal aspects of the roots surfaces [1, 9, 19, 31, 32]. However, two, three, and four-rooted variations are rarely reported in the literature [33-37]. Several studies indicated high level of endodontic failures and flare-ups due to complexity and diversity of root canal configurations in mandibular premolars [38-40]. These facts are confirmed with several studies in mandibular premolars (Table 1). In 2014, Llena *et al.* [41] reported *type I* (78.1%) followed by *type V* (12.3%) as the most frequent types of Vertucci's classification in mandibular first premolars among a Spanish population. In addition, six studies on Chinese population showed *type I* as the most frequent one (86.8% to 54%) [42-47], and in all of them the second common configuration was *type V*; they also mentioned 10% of all teeth being *type IV* and 6% of them was *type II* and *III* [47]. Among Turkish (60.6%) [16], Jordanian (58.2%) [48], Indian (80%, 72%, 67.4%) [49-52] and Egyptian (61.2%) [53] populations, also *type I* was the most frequent one, but the prevalence of the other types of canals were various. In second mandibular premolars among Indian, Jordanian, Turkish, Chinese and Spanish populations like first mandibular premolars, most of root canals were Vertucci's *type I*. Within the other types, most of the root canals were *type V* [16, 41, 43, 44, 48, 49, 52, 54-56]. 

In addition, a wide variety of methods have investigated root canal morphology. There are some laboratory techniques such as clearing technique using decalcification [57] (31) with injection of India ink [58-60], hematoxylin dye [17], Chinese ink [61] or metal castings [62, 63], *in vitro* radiography [12, 13, 64], *in vitro* macroscopic examination [65], grinding or sectioning [18, 66], and scanning electron microscopy (SEM) examination [67]. Moreover, computed tomography (CT), spiral-CT, micro-CT, and cone-beam CT (CBCT) were used for clinicall investigation [4, 68, 69]. All of these methodological and biological factors contribute to variations in the reported prevalence. The current study aimed to review the root canal configurations of root canals in mandibular first and second premolars among Iranian population based on separated epidemiological studies.

## Materials and Methods

A comprehensive search was conducted to retrieve published articles related to root canal configuration and prevalence of each type of root canals based on Vertucci’s classification [17] among root canal of mandibular premolars. An electronic search was conducted in Medline, Scopus and Google Scholar from January 1984 to September 2015 without language limitation publications with available full texts by using the following keywords: “Root Canal Anatomy”, “Root Configuration”, “Root Canal Morphology”, “Mandibular First Premolars” and “Mandibular Second Premolars”. Moreover, similar search strategy was also applied by the Cochrane Database and manual searches, including journals and reference lists. Studies which were not classified configuration types based on Vertucci’s classification or those with no mentioned ethnicity were excluded. A total of 473 studies were found in the preliminary search. Then, titles and abstracts were assessed to determine the appropriate and related articles to the subject of our study. After exclusion of irrelevant studies, 52 articles remained. Then the full texts of the selected articles were obtained and reviewed. In each study we extracted the methodology, sample size, sampling location and prevalence of different types of root canal configuration. Among these studies, 22 articles remained which mentioned these data and their classification based on Vertucci’s classification and only four studies conducted in Iranian population [70-73]. Websites such as *www.iranmedex.com*, *www.magiran.com* and *www.sid.ir* were used to search all concerning studies published in Persian. From total of 31 articles found of this search, seven studies fulfilled the inclusion criteria.

## Results

**Figure 2:**
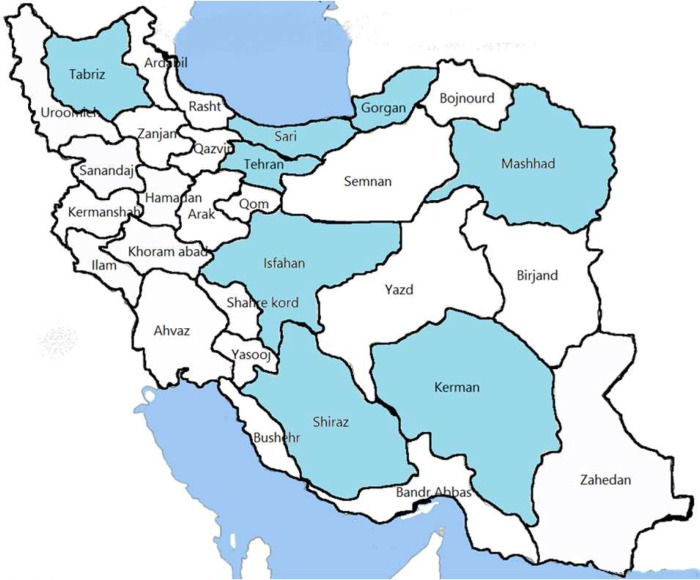
Geographic distribution of the selected studies and prevalence of root canal configurations in mandibular premolars

Eleven studies were done on root canal morphology of mandibular premolars in Iranian population; five investigated both first and second mandibular premolars, four investigated only first mandibular premolars, and two articles were conducted among just second mandibular premolars. In total, 1644 mandibular first premolars and 1268 second premolars were investigated (Table 2 and Figure 2). In all studies, Vertucci’s *type I* classification was the most frequent type of canal configuration among mandibular premolars, except for the study by Sadr Lahijani [74] which was conducted within Kerman province and indicated *type III* as the most frequent (68%) in mandibular first premolars. 

***Mandibular first premolars***

In 2013, Sobhani *et al. *[75] noted that 90.8% of root canals were classified as *type I* while 9.2% were going to other types in Tehran population. In their study CBCT was used and 577 teeth were analyzed. Another study on Gorgan population showed similar results (88.4% *type I*) *via* radiography and sectioning the fuchsine stained teeth [71]. However, in this study the prevalence of *type V* was 4.1% [71] instead of 2.3% in the previous study [75]. In 2007, Rahimi *et al*. [72] in a study on Tabriz population demonstrated the highest prevalence of *type* *V* (16.9%) between reviewed studies. They evaluated 163 teeth using ink injection. Two related studies in Kerman population noted different results in the prevalence [74, 76]. Kouzekanani *et al.* [76] assessed 280 teeth using ink injection and showed *type I* canal configuration as the most common one (79%) followed by *type III* (5.6%), *type II* (5%), *type V* (4.5%), and *type IV* (2.2%). However, Sadr Lahijani *et al.* [74] showed *type III* canal configuration as the most common (64%) followed by *type II* (24 %), *type I* (10%) and *type V* (2%). They also used ink injection for the evaluation but only 50 teeth were assessed. These differences may have occurred due to the low sample size in the second study. Safi *et al.* [77] and Madani *et al*. [78] used stereomicroscope to study on Shiraz and Babol populations, respectively. Their results were in accordance to other studies. In 2005, Hashemi Nia *et al. *[79] indicated the most pevalence of *type III* (11.6%) in canal configuration among Isfahan population. Moreover, Zarrabi *et al.* [80] showed the most percentage of *type IV* (29%) between studies reviewed. They performed the investigation by using stereomicroscope in Mashhad population. 

***Mandibular second premolars***

In 2013, Bolhari *et al.* [70] noted that 91.24% of root canals classified as *type I* and the 8.86% were going to other types in Tehran population. Radiography and fuchsine staining were used to evaluate 217 teeth. In another study conducted among Tehran population, Sobhani *et al.* [75] showed approximately similar results. In this study 611 teeth were assessed by using CBCT. In two studies conducted in Tabriz, Rahimi *et al. *[72, 73] investigated 137 teeth by stereomicroscopy in 2009 and 103 teeth by ink injection in 2007. However, there are some differences between the prevalence. In the second study (2009) they mentioned *type II, type III *and* type IV* more frequent than the same type in the previous study. Moreover, both studies indicated *type I* as the most common configuration. Sadr Lahijani *et al. *[74] showed the most percentage of *type II* (20%) and *type III* (24%) between studies reviewed. In addition, Safi *et al.* [77] reported a 10% prevalence for *type IV* canal configuration which was the most prevalence in the evaluated articles reviewed.

## Discussion

 Knowledge about root canal configuration is essential for all clinicians to accomplish a successful root canal treatment. Variation of root canal configuration, internal and external root anatomy influence the outcome of root canal therapy [25, 26, 81, 82]. The current study assessed the epidemiological studies which investigated root canal morphology of mandibular premolars in several provinces of Iran. In total, out of eleven studies conducted in eight provinces, 1644 mandibular first premolars and 1268 second premolars were investigated. Totally among mandibular first premolars, 70.9% were *type I* of Vertucci's classification, followed by 10.4% *type III*, 7.18% *type IV*, 5.23% *type II* and 5.16% *type V*. In addition, among mandibular second premolars, 82.86% were *type I*, 6.25 *type III*, 5.32% *type II*, 4.27% *type IV* and 0.69% *type V*. These results indicate the importance of seeking for extra canals in both mandibular premolars due to 29.1% probability of not being one root canal in mandibular first premolars and 27.14% of its probability in the second premolars. In accordance to our results, other studies also indicated that mandibular premolars are difficult for root canal therapy due to the diversity and complexity of internal anatomy [38, 47, 83].

***Mandibular*** ***first premolars***

Mandibular first premolars are more commonly single rooted and their routine canal configuration is *type I*. This fact can lead the clinicians to misdiagnosis and failure of treatment due to the possibility of having more than one root canal in Iranian population. These results are in accordance to the reported prevalence within the other populations. There is a frequency of 21.9% of a Spanish population of mandibular first premolars having more than a single canal [41], 20% of an Indian population [49], 38.8% of an Egyptian population [53], 37.5% of a Chinese population [46], 39.4% of Turkish population [16], and 40.8% of a Jordanian population [48]. These results indicated an ethnicity relation and it seems that more epidemiological studies are required to investigate the exact prevalence in each population. 

***Mandibular*** ***second premolars***

Mandibular second premolars are most commonly single-rooted, however the prevalence of having more than one canal (27.14%) within Iranian population is considerable for clinicians. Moreover, there is a frequency of 28% in Jordanian population [48], 29% of a Turkish population [16], 34% of Indian population [49], 9.4% of a Spanish population [41] and 8% of Chinese population [43]. However, it seems frequency of more than one root canal and variations are lesser among mandibular second premolars compared to the first premolars [81, 86]. 

**Table 1:** Studies included in this systematic review and their related prevalence of root canal configurations [Vertucci’s type (%)] in mandibular premolars worldwide (SS: sample size, N/M: not mentioned

	**Investigator **	**Method of study**	**Race**	**SS **	***Type I***	***Type II ***	***Type III***	***Type IV ***	***Type V ***
**First Premolars**	**Llena ** ***et al.*** ** [** 41 **]**	CBCT	Spanish	73	78.1	8.2	0	0	12.3
	**Singh and Pawar [** 49 **]**	Indian ink	Indian	100	80	6	0	10	2
	**Alhadainy [** 53 **]**	Black ink	Egyptian	250	61.2	5.6	2.8	13.2	16.4
	**Liu ** ***et al*** **. [** 46 **]**	Micro CT	Chinese	113	62.5	0	3	0	26
	**Yang ** ***et al. *** **[** 45 **]**	CBCT	Chinese	238	76.14	3.14	2.73	6.59	9.32
	**Yu ** ***et al.*** ** [** 44 **]**	CBCT	Chinese	178	86.8	0	3	0	17
	**Tian ** ***et al*** **.[** 43 **] **	CBCT	Chinese	178	86.8	0	1.7	0	9.8
	**Jain and Bahuguna[** 50 **]**	Clearing	Indian	138	67.4	8	3.7	3.9	17.4
	**Liao ** ***et al.*** ** [** 42 **]**	CBCT	Chinese	97	83.5	0	3.6	0	8.8
	**Velmurugan and Sandhya [** 51 **]**	Oil based dye	Indian	100	72	6	3	10	8
	**Awawdeh and Al-Qudah [** 48 **] **	Indian ink	Jordanian	500	58.2	4.8	1.4	14.4	16.8
	**Lu ** ***et al*** **. [** 47 **] **	Methylene blue	Chinese	44	54	6	6	10	0
	**Sert and Bayirli [** 16 **]**	Clearing	Turkish	200	60.6	18.5	10.5	7	2.5
	**Sikri and Sikri [** 52 **]**	Spiral CT	Indian	112	80	9	3	2	4
	**Baisden ** ***et al*** **. [** 84 **] **	Hematoxylin dye	N/M	106	76	0	0	24	0
	**Vertucci [** 56 **]**	Hematoxylin dye	N/M	400	70	4	1.5	24	0.5
**Second Premolars**	**Llena ** ***et al*** **. [** 41 **]**	CBCT	Spanish	53	90.6	1.6	0	0	7.5
	**Singh and Pawar [** 49 **]**	Indian ink	Indian	100	66	30	0	0	4
	**Yu ** ***et al.*** ** [** 44 **]**	CBCT	Chinese	178	97.2	0.55	0	0	3
	**Tian ** ***et al*** **. [** 43 **] **	CBCT	Chinese	178	92	0	0	0.5	1.69
	**Parekh ** ***et al*** **. [** 54 **]**	Clearing	Indian	40	80	0	0	2.5	17.5
	**Awawdeh and Al-Qudah [** 48 **] **	Indian ink	Jordanian	400	72	3.8	1	7.5	15.3
	**Sert and Bayirli [** 16 **]**	Clearing	Turkish	200	71	7	3.5	9	7
	**Caliskan ** ***et al*** **. [** 55 **] **	Clearing	Turkish	100	93.7	0	0	0	6.4
	**Sikri and Sikri [** 52 **] **	Spiral CT	Indian	96	80	9	3	2	4

**Table 2.** Studies included in this review and their related prevalence of root canal configurations [Vertucci’s type (%)] in mandibular premolars among Iranian population (SS: sample size

	**Investigator **	**Method of study**	**City**	**SS **	***Type I***	***Type II ***	***Type III***	***Type IV ***	***Type V ***
**First Premolars**	**Sobhani ** ***et al.*** ** [** 75 **]**	CBCT	Tehran	577	90.8	1.9	3.4	1.6	2.3
	**Khedmat ** ***et al*** **. [** 71 **]**	RG and fuchsine	Gorgan	217	88.4	1.8	3.2	0.9	4.1
	**Rahimi ** ***et al*** **. [** 72 **]**	Indian ink	Tabriz	163	70.6	1.9	3.8	3.8	16.9
	**Kouzekanani ** ***et al*** **. [** 85 **]**	Indian ink	Kerman	280	79	5	5.6	2.2	4.5
	**Sadr Lahijani ** ***et al*** **. [** 74 **] **	Indian ink	Kerman	50	10	24	64	2	0
	**Safi ** ***et al*** **. [** 77 **]**	Stereomicroscope	Shiraz	50	90	0	2	8	0
	**Hashemi Nia ** ***et al*** **. [** 79 **]**	Indian ink	Isfahan	127	67	4.5	11.6	14.2	2.7
	**Madani ** ***et al*** **. [** 78 **] **	Stereomicroscope	Babol	100	86	2	0	3	8
	**Zarrabi ** ***et al*** **. [** 80 **]**	Stereomicroscope	Mashhad	100	57	6	0	29	8
	**Mean of total**			1644	70.9	5.23	10.4	7.18	5.16
**Second Premolars**	**Bolhari ** ***et al*** **. [** 70 **]**	RG and fuchsine	Tehran	217	91.24	3.22	1.84	1.38	1.38
	**Sobhani ** ***et al*** **. [** 75 **]**	CBCT	Tehran	611	90.7	2.7	3.1	2	1.5
	**Rahimi ** ***et al*** **. [** 73 **]**	Stereomicroscope	Tabriz	137	89.79	1.46	2.92	3.64	0
	**Rahimi ** ***et al*** **. [** 72 **]**	Indian ink	Tabriz	103	76.3	7.9	9.9	5.9	0
	**Sadr Lahijani ** ***et al*** **. [** 74 **] **	Indian ink	Kerman	50	54	20	24	2	0
	**Safi ** ***et al*** **. [** 77 **]**	Stereomicroscope	Shiraz	50	88	0	2	10	0
	**Madani ** ***et al*** **. [** 78 **] **	Stereomicroscope	Babol	100	90	2	0	5	2
	**Mean of total**			1266	82.86	5.32	6.25	4.27	0.69

These differences in frequencies may be due to various factors. Trope *et al.* [36] in 1986 investigated the role of ethnicity in the frequency of different root canal configurations and demonstrated that African Americans show more tendency to having more than one root canal compared to Caucasians. In addition, several studies all around the world indicated racial relation of root canal morphology [87-91]. Sert and Bayirli [16] demonstrated the differences related to gender in a Turkish population. Their results showed higher prevalence of more than one root canal in female patients. 

Moreover, the other possible factor are the various methods that were used for the assessment of internal anatomy. Clearing technique is used frequently [92-94] for the evaluation; however, it has limitation in diagnosis of C-shaped canals and can only be applied after extraction [95]. Conventional radiographic evaluation does not seem reliable enough based on the literature which compared it to clearing technique [47, 96, 97]. However, new approaches such as micro-CT and CBCT are considered as reliable as the clearing technique and dye injection mainly for research purposes [98, 99]. In practice, it seems that application of pre-curved stainless steel files and tactile sense can be helpful for clinicians to determine the presence of additional canal(s) [39]. In addition, different studies indicated that different radiographies with various horizontal angulation can also be useful for practitioners [100, 101].

## Conclusion

Our review demonstrated that most of mandibular premolars have a single root canal (70.9% of first premolars and 82.86% of second premolars) in Iranian population. These results highlight that clinicians should be aware of failures due to extra missed canals and avoid them. Moreover, ethnical characteristics of premolars in Iranians can be observed/documented.
